# Neonatal outcome of dicephalus parapagus twins with a shared heart: A case report and review of prognostic factors

**DOI:** 10.1016/j.ijcchd.2026.100723

**Published:** 2026-10-29

**Authors:** Hanan Alsehli, Saif Alsaif

**Affiliations:** a King Saud Bin Abdulaziz University for Health Sciences, Riyadh, Saudi Arabia; b Department of Obstetrics and Gynecology, King Abdulaziz Medical City, Ministry of the National Guard-Health Affairs, Riyadh, Saudi Arabia; c King Abdullah International Medical Research Centre, Riyadh, Saudi Arabia; d Department of Neonatal Intensive Care Unit, King Abdulaziz Medical City, Ministry of the National Guard-Health Affairs, Riyadh, Saudi Arabia

**Keywords:** Conjoined twin, Dicephalus, Parapagus twins, Monoamniotic twins, Neonatal outcome

## Abstract

Conjoined twins are a rare type of monoamniotic twins, occurring in 1.47 per 100,000 births worldwide. Prognosis depends on the extent of organ sharing, particularly the heart. We report a case of parapagus dicephalus tribrachius dipus conjoined twins diagnosed at 19 weeks in a 24-year-old Saudi woman. Ultrasound showed twins with two heads, four upper limbs, two lower limbs, and sharing major organs. Fetal echocardiography showed one structurally normal heart and another small heart with a single outflow tract. A cesarean section was performed at 33 weeks of gestation, delivering cyanosed twins who died 53 minutes after birth. Parapagus dicephalus twins are among the most common forms of conjoined twins and carry a high risk of perinatal mortality, especially when a single functional heart is shared. Early prenatal diagnosis, multidisciplinary counseling, and discussion of management options, including termination when appropriate, are crucial for guiding parental decisions and optimizing care.

## 1 Introduction

Conjoined twins represent a rare type of monoamniotic twins, with an estimated incidence of 1.47 per 100,000 births worldwide. The degree of fusion between the anatomical structures of one twin and the co-twin varies, ranging from minor to substantial. The location of fusion categorizes it as cephalopagus, thoracopagus, omphalopagus, ischiopagus, parapagus, craniopagus, rachipagus, and pygopagus [1]. A stillbirth occurs in approximately 50% of conjoined twin pregnancies. Additionally, 35% of conjoined twins die within 24 hours, and 15% to 18% survive from prenatal ultrasound diagnosis to successful separation [2], [3]. It is hypothesized that conjoined twins are the product of abnormal embryogenesis. Fusion and fission are the two primary theories suggested for explaining the phenomenon. The prognosis and outcome of a separation surgery depend on the degree of organ sharing, particularly in the heart [4].

Understanding the expected outcome of a specific type of conjoined twin can aid in counseling the parents. This case report and review focus on the outcomes of dicephalus parapagus conjoined twins who share one heart.

## 2 Case presentation

A 24-year-old primigravid Saudi woman was referred from a primary hospital in Alzulfi City to the Women's Health Hospital in Riyadh with a diagnosis of conjoined twins sharing a single pelvis at 19 weeks and 6 days of gestation. The pregnancy was spontaneous. She had no medical or surgical history, and she denied any family history of fetal anomalies, genetic disorders, or multiple pregnancies. An anatomy ultrasound revealed conjoined twins with two heads, four upper limbs, and two lower limbs; they shared one stomach, one liver, two kidneys, and one urinary bladder (Fig. 1). Assessment of the fetal heart during the anatomy scan was challenging. Fetal echocardiography showed that one twin had a structurally normal heart, while the other had a small heart with a single unobstructed outflow tract, a small aortic arch, and a small ductal arch, with no visible intracardiac anatomy. Fetal MRI demonstrated parapagus dicephalus tribrachius dipus conjoined twins with separate heads, faces, and spines. They shared a thoracic cage and possibly had separate lungs. They also shared a diaphragm, with evidence of congenital diaphragmatic hernia or eventration containing the liver. The twins shared a single abdominal cavity, two lower limbs, two upper limbs, and a third upper limb arising proximally from the medial aspect, with separate hands distally. Amniocentesis with FISH analysis revealed a normal female karyotype (46,XX) with no evidence of aneuploidy.

**Fig. 1:**
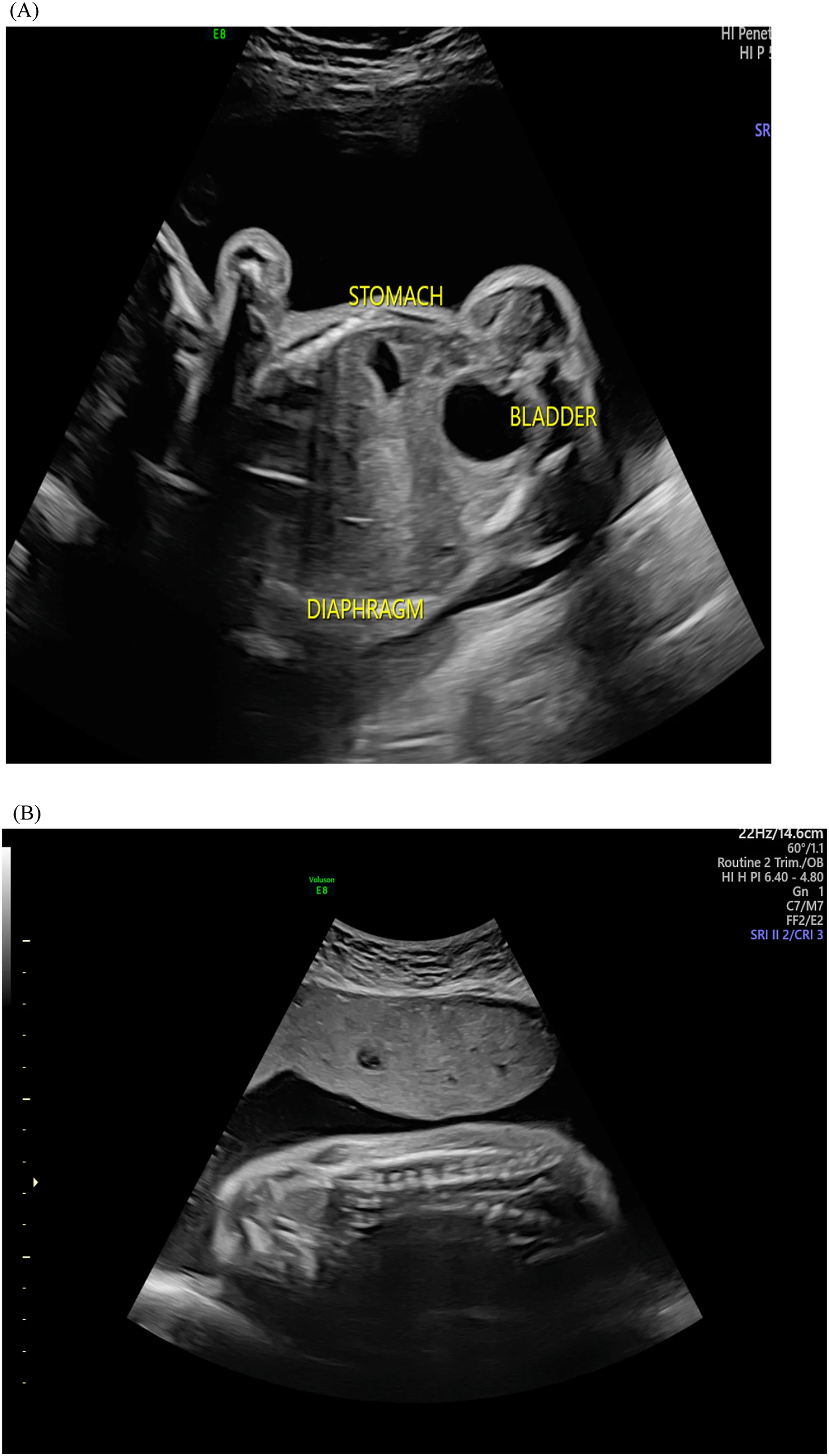
Two-dimensional ultrasound images of conjoined twins showing two heads, four upper limbs, and two lower limbs. The twins share one stomach, one liver, two kidneys, and one urinary bladder.

The parents were counseled by a multidisciplinary team consisting of a maternal-fetal medicine consultant, a neonatal intensive care consultant, a pediatric surgeon, and a social worker. The fetal and neonatal prognosis was discussed, and due to the poor expected outcome, the team recommended comfort care, with which the parents agreed. An elective cesarean section was performed at 33 weeks of gestation. The twins were delivered through a transverse skin incision and a lower transverse uterine segment incision without complications or extension of the uterine incision. The outcome was conjoined twins with separate heads, faces, spines, and right arms; a shared thoracic cage and left upper arm with a separate hand; a single abdominal cavity; two lower extremities; female genitalia; and a single anus (Fig. 2). At birth, the twins were cyanosed. Their initial heart rate was 70 beats per minute, which gradually decreased to asystole, leading to neonatal death 53 minutes after delivery. Postnatal chromosomal analysis confirmed a 46, XX karyotype with no evidence of aneuploidy. Skeletal X-ray demonstrated parapagus conjoined twins with shared thoracic and abdominal cavities, separate spinal vertebrae, a shared pelvis, two lower extremities, and fused upper extremities.

**Fig. 2:**
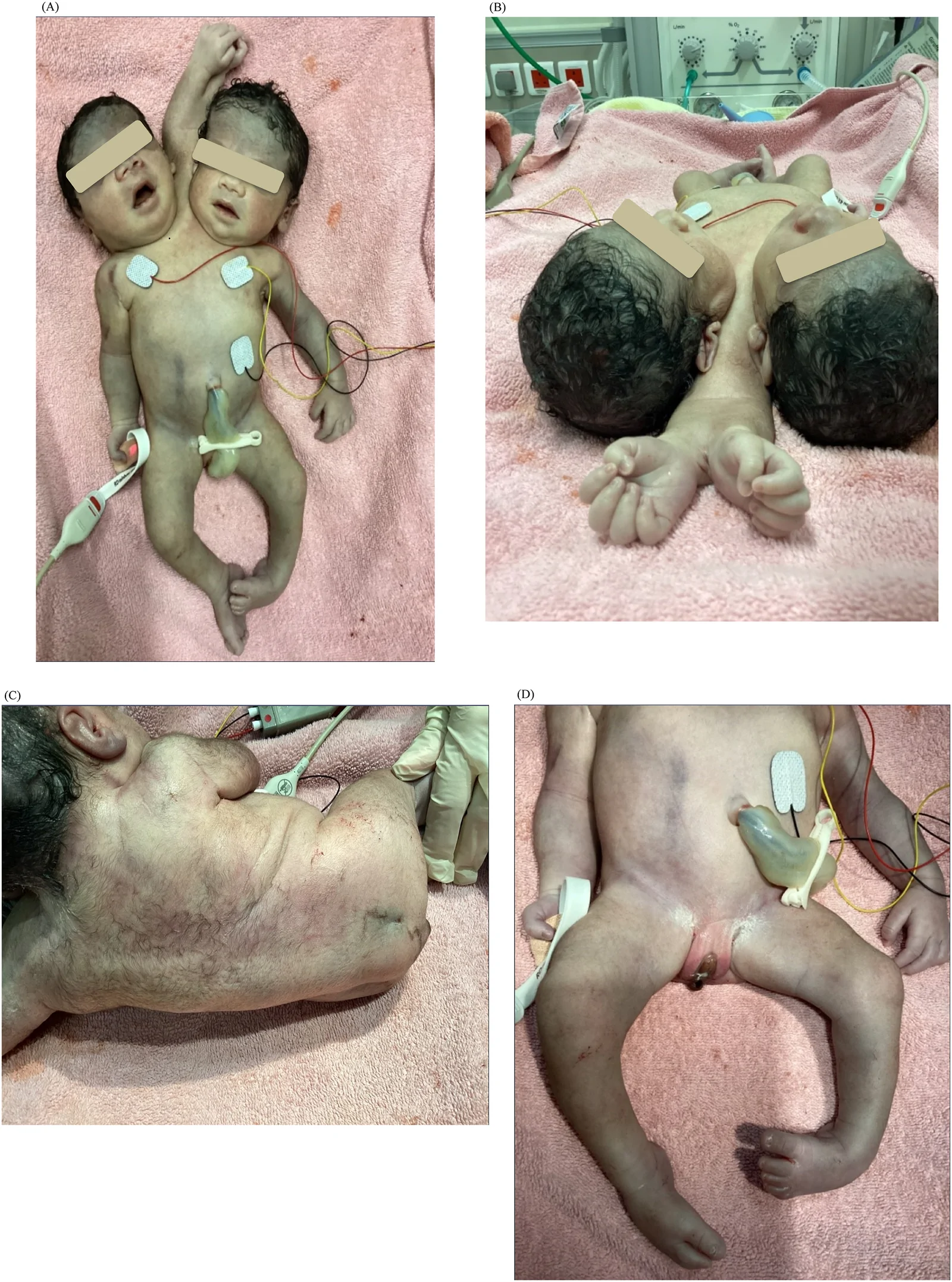
Postnatal images of conjoined twins showing distinct and shared anatomical features.

## 3 Discussion

Conjoined twins represent a rare type of monoamniotic twins. They are more prevalent in females, with a male-to-female ratio of 1:3 [4]. The most prevalent type of conjoined twin is the thoracopagus type, with 42.0% of cases reported. Parapagus dicephalus was the second most prevalent conjoined twins type, accounting for 11.5% of the total reported cases [1].

Dicephalus twins have a very high stillbirth and mortality rate [5], [6]. Congenital anomalies and the extent of organ sharing significantly influence survival prospects. Numerous case reports have documented the fetal outcome, revealing the following: In a previous report, a baby of a dicephalus parapagus twin with a fused thorax died 1 h after being delivered with a J-shaped uterine incision at 28 weeks [7]. Similarly, twins with a shared single heart delivered at 37 weeks by emergency cesarean section survived for only 15 minutes [8]. Another report documented the survival of dicephalus conjoined twins, born at 36 weeks with a single large heart, for three days [9]. A retrospective review of 14 sets of conjoined twins revealed two cases of dicephalus tribrachius dipus, the first of which had two separate hearts. Twin A had a normal heart, while twin B had a large VSD and ASD and a possible tetralogy of Fallot. A cesarean section was performed at 36 weeks. However, the smaller twin's cyanotic heart disease was incompatible with life without separation, leading to their death shortly after birth. The other twin, who had a shared abnormal heart, underwent a cesarean section at 34 weeks of gestation and died shortly after birth [5]. Another case report described twins with a single heart delivered at 36 weeks who survived for 28 hours [10] (Table 1). While preterm birth increases neonatal mortality, most reported cases involved late preterm births (34–37 weeks), where survival rates tend to be higher compared to early preterm births (<32 weeks) [11]. However, in these instances, the mortality is associated with the shared heart. The most important organ that can determine survival is sharing the same heart; all previously reported cases showed a high neonatal mortality with a shared single heart. These results highlight that neonatal survival in dicephalus parapagus twins is significantly affected by the existence of a shared heart, with mortality rates remaining elevated irrespective of gestational age.

**Table 1:** Reported outcomes of dicephalus parapagus twins with a shared heart.

**Reference**	Gestational Age at Delivery	Delivery Mode	Cardiac Anatomy	Survival Duration
7	28 weeks	Cesarean (J-shaped uterine incision)	Shared heart (fused thorax)	1 hour
(8)	37 weeks	Emergency Cesarean	Shared single heart	15 minutes
(9)	36 weeks	Not specified	Shared large heart	3 days
(5) – Case 1	36 weeks	Cesarean	Two separate hearts (Twin B with complex CHD: VSD, ASD, possible TOF)	Died shortly after birth
(5) – Case 2	34 weeks	Cesarean	Shared abnormal heart	Died shortly after birth
(10)	36 weeks	Not specified	Shared single heart	28 hours

Congenital anomalies are three-to fivefold more prevalent in monozygotic twins than in dizygotic twins, and they are more common in monozygotic monochorionic twins compared to monozygotic dichorionic twins [12], [13], [14]. Previous meta-analyses indicated that monochorionic/diamniotic twin gestation is a risk factor for congenital heart defects [15]. In this case, we observed anomalies involving twins who shared a thoracic cage and may have had separate lungs. They also shared a diaphragm, which exhibited signs of a congenital diaphragmatic hernia or eventration that included the liver. One twin had a structurally normal heart, while the other had a smaller heart. Spencer's study confirms the previous findings, demonstrating that Siamese twins originate from two independent notochords, with the original intention of developing as separate twins. These twins fuse due to proximity, forming anterolaterally. They share a heart, liver, and diaphragm; have separate respiratory and upper gastrointestinal tracts; and have a single shared genitourinary system [16]. Our case did not present with any diagnosed neural tube defects, in contrast to a previous report that indicated over 15% of parapagus dicephalus cases had such defects [1].

Our case revealed a normal karyotype result, consistent with previous reports indicating that chromosomal abnormalities are largely absent in conjoined twins [5]. A previous prospective study, which included 495 twin pregnancies, diagnosed a chromosomal abnormality in six fetuses (0.6%), all from dichorionic pregnancies [13].

## 4 Conclusion

Parapagus dicephalus twins are among the most frequently reported forms of conjoined twinning and are associated with an elevated rate of perinatal mortality and stillbirth, particularly when a single functional heart is shared. A shared heart is a critical determinant of poor neonatal outcomes. Comprehensive multidisciplinary counseling, early prenatal diagnosis, and the discussion of all management options, including pregnancy termination when legally and ethically appropriate, are essential for guiding parental decision-making and preparing for postnatal care.

## CRediT authorship contribution statement

**Hanan Alsehli:** Writing – review & editing, Writing – original draft. **Saif Alsaif:** Writing – review & editing.

## Ethics declaration

Written informed consent to take part in the study and to publish the article has been obtained from all participants or their legal representatives. The privacy rights of participants have been observed.

This study was performed in compliance with relevant laws, regulatory frameworks and guidelines where the research took place. This study was approved by the King Abdullah International Medical Research Center's (KAIMRC) institutional review board. (Approval No. IRB/NRR. 25/137/7)

This research follows the CARE guidelines and the CARE checklist.

## Consent for publication

This case report and any accompanying images were published with the parents' written informed approval. The Editor-in-Chief of the journal has access to a copy of the written consent for review.

## Availability of data and materials

The datasets used and/or analyzed during the current study are available from the corresponding author on reasonable request.

## Code availability

Not applicable.

## Declaration of generative AI and AI-assisted technologies in the manuscript preparation process

AI was not used during the preparation of this manuscript.

## Funding statement

There was no specific funding obtained for this study.

## Declaration of competing interest

The authors declare that they have no known competing financial interests or personal relationships that could have appeared to influence the work reported in this paper.
